# Rituximab therapy reduces activated B cells in both the peripheral blood and bone marrow of patients with rheumatoid arthritis: depletion of memory B cells correlates with clinical response

**DOI:** 10.1186/ar2798

**Published:** 2009-08-28

**Authors:** Magda Nakou, Georgios Katsikas, Prodromos Sidiropoulos, George Bertsias, Eva Papadimitraki, Amalia Raptopoulou, Helen Koutala, Helen A Papadaki, Herakles Kritikos, Dimitrios T Boumpas

**Affiliations:** 1 Department of Rheumatology, Clinical Immunology and Allergy, University of Crete, Medical School, Voutes 71500, Heraklion, Greece; 2 Laboratory of Flow Cytometry, University of Crete, Medical School, Voutes 71500, Heraklion, Greece; 3 Department of Hematology, University of Crete, Medical School, Voutes 71500, Heraklion, Greece

## Abstract

**Introduction:**

Bone marrow (BM) is an immunologically privileged site where activated autoantibody-producing B cells may survive for prolonged periods. We investigated the effect of rituximab (anti-CD20 mAb) in peripheral blood (PB) and BM B-cell and T-cell populations in active rheumatoid arthritis (RA) patients.

**Methods:**

Active RA patients received rituximab (1,000 mg) on days 1 and 15. PB (n = 11) and BM (n = 8) aspirates were collected at baseline and at 3 months. We assessed B-cell and T-cell populations using triple-color flow cytometry.

**Results:**

Rituximab therapy decreased PB (from a mean 2% to 0.9%, *P *= 0.022) but not BM (from 4.6% to 3.8%, *P *= 0.273) CD19^+ ^B cells, associated with a significant reduction in the activated CD19^+^HLA-DR^+ ^subset both in PB (from 55% to 19%, *P *= 0.007) and in BM (from 68% to 19%, *P *= 0.007). Response to rituximab was preceded by a significant decrease in PB and BM CD19^+^CD27^+ ^memory B cells (*P *= 0.022). These effects were specific to rituximab since anti-TNF therapy did not reduce total or activated B cells. Rituximab therapy did not alter the number of activated CD4^+^HLA-DR^+ ^and CD4^+^CD25^+ ^T cells.

**Conclusions:**

Rituximab therapy preferentially depletes activated CD19^+^HLA-DR^+ ^B cells in the PB and BM of active RA patients. Clinical response to rituximab is associated with depletion of CD19^+^CD27^+ ^memory B cells in PB and BM of RA patients.

## Introduction

Rheumatoid arthritis (RA) is a complex inflammatory autoimmune disease characterized by disturbances in T-cell and B-cell functions. Clinical and animal studies highlight the multiple roles of B cells in the development and severity of RA, including production of autoantibodies, inflammatory cytokines such as TNF and IL-6, and aberrant antigen presentation [1]. Recent data in animal models suggest that, among these effects, the antigen-presenting capacity of B cells may be of particular importance in RA pathogenesis [2].

Rituximab is a chimeric mAb against CD20 that induces a profound depletion of B cells in the peripheral blood of RA patients [3]; however, little is known about the qualitative and quantitative aspects of deletion achieved in tissues including the bone marrow (BM) and lymph nodes. Initial studies in humans with RA suggest that B cells are also depleted in the BM as well as in the synovium [4]; nevertheless, the depletion is rather incomplete [5].

The BM is important for the biology of B cells as it represents a site of B-cell differentiation and maturation. The BM is an immunologically privileged site, where stroma promote B-cell survival and thus may protect B cells from depleting therapies [6]. We have previously described quantitative and qualitative changes in the BM of RA patients, which could affect a variety of BM resident cells including the B cells [7,8]. Furthermore, based on gene expression studies of lupus patients, we have reported that the BM may be more informative than peripheral blood (PB) in differentiating active from inactive lupus patients and in differentiating patients from control individuals [9]. In the present article we explored the effect of rituximab treatment on B-cell subpopulations in the periphery and in the BM in a cohort of RA patients with resistant disease.

## Materials and methods

### Patients and treatment

Thirty-one RA patients with active disease (disease activity score of 28 joint counts (DAS28) >5.1) despite treatment with disease-modifying anti-rheumatic drugs including at least one anti-TNF agent, were selected to receive rituximab. Patients were followed in the Department of Rheumatology, Clinical Immunology, and Allergy, University Hospital of Heraklion (Greece).

PB and BM specimens were obtained from 11 consenting patients without predefined selection criteria. Samples were obtained at baseline and after 12 weeks of treatment. Rituximab was administered as a 1,000 mg intravenous infusion on days 1 and 15 [3]. Seven RA patients starting anti-TNF agents were used as controls for the immunological study. Patients had not received steroids for at least 24 hours before PB and BM sampling. Written informed consent was obtained from all patients and healthy controls, and the study was approved by the Ethics Committee of the University Hospital of Heraklion.

### Clinical assessment

Clinical parameters (28 swollen and tender joint counts), functional status (Health Assessment Questionnaire), and laboratory parameters were regularly assessed every 2 months. The DAS28 was applied to assess clinical efficacy [10]. The European League Against Rheumatism response criteria based on DAS28 were used to assess the response to therapy [11].

### Isolation of mononuclear cells and flow cytometry

PB mononuclear cells and BM mononuclear cells were isolated by Ficoll-Histopaque (Sigma-Aldrich, St Louis, MO, USA) density-gradient centrifugation of heparinized samples. Cells (5 × 10^5^) were stained with the appropriate amounts of fluorochrome-conjugated monoclonal antibodies for 30 minutes on ice. Combinations of anti-CD19, anti-HLA-DR, anti-CD27, anti-CD38 and anti-CD45 staining were used for analysis of B cells. For analysis of T cells, anti-CD3, anti-CD4, anti-HLA-DR, and anti-CD69 staining was performed. IgG isotype controls were used in all experiments to determine the positively-stained cell population. Antibodies to CD19, CD69, CD27, CD45, and CD38 were purchased from Immunotech (Marseille, France), and anti-HLA-DR, anti-CD4, anti-CD3 antibodies were purchased from eBioscience (San Diego, CA, USA).

At least 200,000 events were collected in the lymphocyte gate for analysis in an Epics Elite flow cytometer (Coulter, Miami, FL, USA). Lymphocyte subsets were defined as follows: naïve B cells, CD19^+^CD27^-^; memory B cells, CD19^+^CD27^+^; activated B cells, CD19^+^HLA-DR^+^; activated T cells, CD4^+^HLA-DR^+^, CD4^+^CD25^+^, CD4^+^CD69^+^. Owing to the low proportion of CD19^+ ^cells, all analyses were performed in constant numbers of CD19^+^-gated cells.

### Statistical analysis

The Statistical Package for Social Sciences (version 16.0; SPSS Inc., Miami, FL, USA) was used in all analyses. Comparisons between independent groups were performed using Student's *t *test, and paired samples were compared using the paired *t *test. The nonparametric Wilcoxon signed-ranks test was used for small (n < 10) sample sizes. Two-tailed *P *< 0.05 was considered statistically significant. Data are presented as mean ± standard error of the mean. Absolute numbers of CD19^+ ^B cells were calculated based on the total lymphocyte numbers in the patient's complete blood count test results, according to the following equation:



## Results

### Clinical characteristics of the patients and response to rituximab therapy

The clinical and laboratory characteristics of the RA patients who were treated with rituximab are summarized in Table 1. Patients had longstanding (mean disease duration = 15 years) and highly active disease (mean DAS28 = 6.3). All but one patient had received at least one anti-TNF agent. Treatment with rituximab reduced disease activity during the first 6 months; response rates (good and moderate) according to the European League Against Rheumatism criteria were 45% at 4 and 6 months. Patient's baseline demographic characteristics, disease activity indexes (DAS28, swollen/tender joint counts, patient's perception of disease activity/pain, functional status), rheumatoid factor levels and erythrocyte sedimentation rate and C-reactive protein values were comparable between responders and nonresponders (data not shown). Rheumatoid factor levels after 4 months of treatment significantly decreased from 253 ± 84 IU/l to 153 ± 80 IU/l (*P *= 0.001, data not shown).

**Table 1:** Demographic and clinical characteristics of rheumatoid arthritis patients treated with rituximab

Age (years)	62 (1.8)
Female (%)	61
Disease duration (years)	15 (2.8)
Previous therapies	
Number of disease-modifying anti-rheumatic drugs used	2.6 (1 to 5)
Number of biologic agents used	1.6 (0 to 3)
Baseline disease characteristics	
Rheumatoid factor-positive (%)	52
Disease activity score of 28 joint counts	6.3 (0.2)
Swollen joints (28 joints)	9.3 (1.1)
Tender joints (28 joints)	12.8 (1.3)
Patient's pain assessment	69.7 (2.9)
Patient's global assessment	67.3 (3.3)
Physician's global assessment	54.8 (3.4)
Health Assessment Questionnaire	0.9 (0.09)
Erythrocyte sedimentation rate (mm/hour)	51 (6.1)
Hemoglobin (g/dl)	12.9 (0.4)
Concomitant disease-modifying anti-rheumatic drugs	
Methotrexate	
Patients (%)	68
Dose (mg/week)	16.3 (7.5 to 25)
Leflunomide	
Patients (%)	29
Dose (mg/day)	17 (10 to 20)
Glucocorticoids	
Patients (%)	29
Dose (mg/day)	5.5 (5 to 10)
Rituximab monotherapy	3

Data expressed as mean (standard error of the mean), percentage, or mean (range); n = 31.

### Rituximab therapy depletes B cells in peripheral blood, but not bone marrow, of RA patients

Immunological studies were performed in 11 consenting patients whose demographic and clinical characteristics were comparable with the remaining patients who received rituximab. We first assessed the effects of rituximab on B cells in RA patients 3 months after therapy.

**Figure 1:**
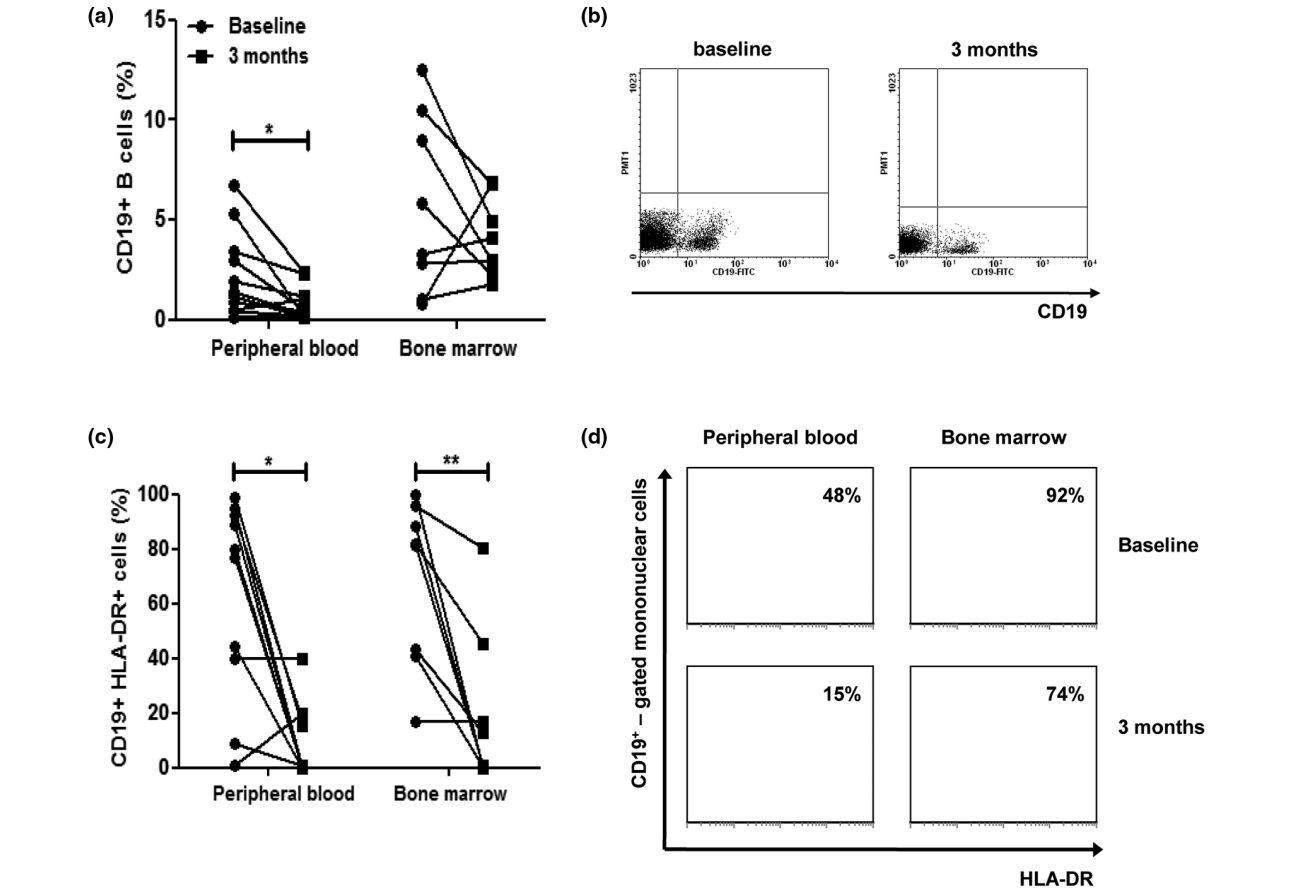
Effect of rituximab on B cells in rheumatoid arthritis peripheral blood and bone marrow. Rituximab preferentially depletes B cells in peripheral blood (PB) and reduces activated B cells in the PB and the bone marrow (BM) of rheumatoid arthritis (RA) patients. **(a) **The percentage of PB (n = 11) CD19^+ ^B cells was significantly reduced following 3 months of treatment with rituximab. A nonsignificant reduction in B cells was also observed in the BM (n = 8) of RA patients. **(b) **Representative flow cytometry analysis of PB CD19^+ ^B cells in a RA patient at baseline (left) and after 12 weeks of rituximab treatment (right). **(c) **Rituximab depletes activated CD19^+^HLA-DR^+ ^B cells both in the PB and in the BM of RA patients. **(d) **Representative flow cytometry histograms of HLA-DR expression in PB and BM CD19^+^-gated cells of a RA patient at baseline and after treatment with rituximab. *P < 0.05 for paired analysis, **P < 0.05.

The percentage of PB CD19^+ ^B cells significantly decreased in all patients from 2.2 ± 0.7% to 0.8 ± 0.3% (*P *= 0.022) (Figure 1a, b). In seven out of 11 patients (64%), complete (>97%) B-cell depletion was noted (Figure 1a). Accordingly, absolute B-cell numbers decreased from 58 ± 12 cells/μl to 17 ± 6 cells/μl (*P *= 0.030) (data not shown). In the BM, however, the effect of rituximab on B cells was less pronounced. Rituximab therapy caused a reduction - but not depletion - of BM B cells in only five RA patients, and overall the mean percentage of BM B cells was not reduced (from 4.6 ± 1.8% to 3.8 ± 0.7%, *P *= 0.273) (Figure 1a). We found no correlation between depletion of B cells in PB or in BM and the clinical response to rituximab. Taken together, these results suggest that the BM B cells are more resistant than PB B cells to anti-CD20 mAb depleting therapy in patients with RA.

### Rituximab preferentially depletes activated CD19^+^HLA-DR^+ ^cells in peripheral blood and bone marrow of RA patients

A key feature in RA pathogenesis is the aberrant antigen-presenting capacity of B cells, contributing to chronic T-cell activation and perpetuation of inflammation in the joint. We therefore assessed the effect of rituximab therapy on B-cell HLA-DR expression, a marker of activation and antigen-presenting function. The proportion of HLA-DR^+ ^cells within the CD19^+ ^cell fraction in PB significantly decreased from 57 ± 12% to 18 ± 9% (*P *= 0.05) (Figure 1c, d). In contrast to the total numbers of CD19^+ ^cells, a comparable reduction in the percentage of HLA-DR^+ ^cells within the CD19^+ ^cell fraction was observed in the BM of RA patients who received rituximab (from 69 ± 11% to 20 ± 10%, *P *= 0.007) (Figure 1c). Rituximab therefore effectively and preferentially depletes activated HLA-DR^+ ^B cells both in the PB and the BM of RA patients.

### Favorable response to rituximab therapy preceded by a decrease in CD19^+^CD27^+ ^memory B cells in peripheral blood and bone marrow of RA patients

**Figure 2:**
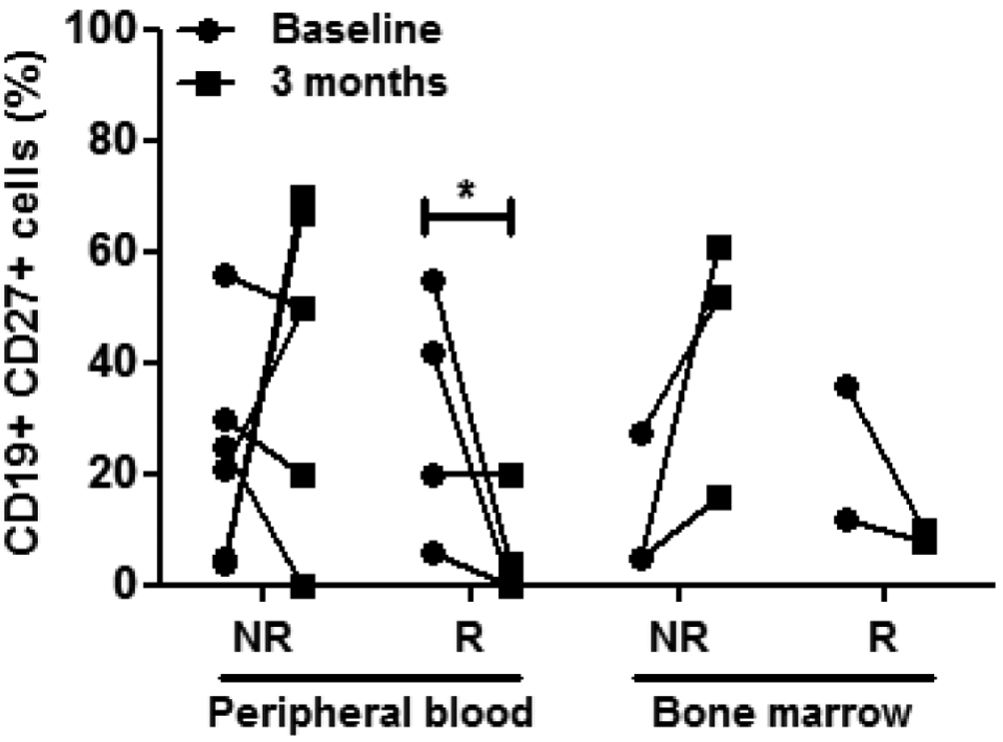
Correlation of clinical response to rituximab with depletion of CD19^+^CD27^+ ^memory B cells. Clinical response to rituximab correlates with depletion of CD19^+^CD27^+ ^memory B cells in peripheral blood (PB) and in the bone marrow (BM) of rheumatoid arthritis (RA) patients. PB CD19^+^CD27^+ ^cells increased by 29 ± 16% in nonresponders (NR) (n = 5), compared with a reduction by 26 ± 10% in responders (R) (*P *= 0.023) (n = 4). Similarly, BM CD19^+^CD27^+ ^cells increased by 31 ± 13% in NR (n = 4), whereas they decreased by 26 ± 13% in R (n = 2). *P < 0.05 for paired analysis between baseline and 3 months.

We next examined the effect of rituximab therapy on memory (CD19^+^CD27^+^) B cells, the precursors of autoantibody-producing plasma cells. The mean percentage of memory B cells in RA patients did not change (26 ± 5% at baseline vs. 26 ± 9% at 12 weeks; data not shown); accordingly, absolute numbers of CD27^+ ^B cells did not differ between baseline and 3 months post-treatment (11 ± 5 cells/μl vs. 6 ± 3 cells/μl). Nevertheless a differential effect was observed according to clinical response to therapy. Patients with a moderate-to-good response to rituximab at 6 months (n = 4) therefore had a preceding significant decrease in PB CD19^+^CD27^+ ^B cells at 12 weeks from 30 ± 7% to 9 ± 5% (*P *= 0.022) (Figure 2). In contrast, in patients who did not respond to rituximab, an increase in PB memory B cells was observed (from 23 ± 8% to 39 ± 13%). Interestingly, similar changes were found in BM CD19^+^CD27^+ ^memory B cells; these B cells decreased by 26 ± 10% in responders (n = 2) but increased by 31 ± 13% in nonresponders (n = 4). Taken together these data suggest that response to rituximab therapy is preceded by a significant decrease in the population of memory B cells both in the PB and in the BM of RA patients.

### Depletion of total and activated B cells is a specific effect of rituximab therapy

**Figure 3:**
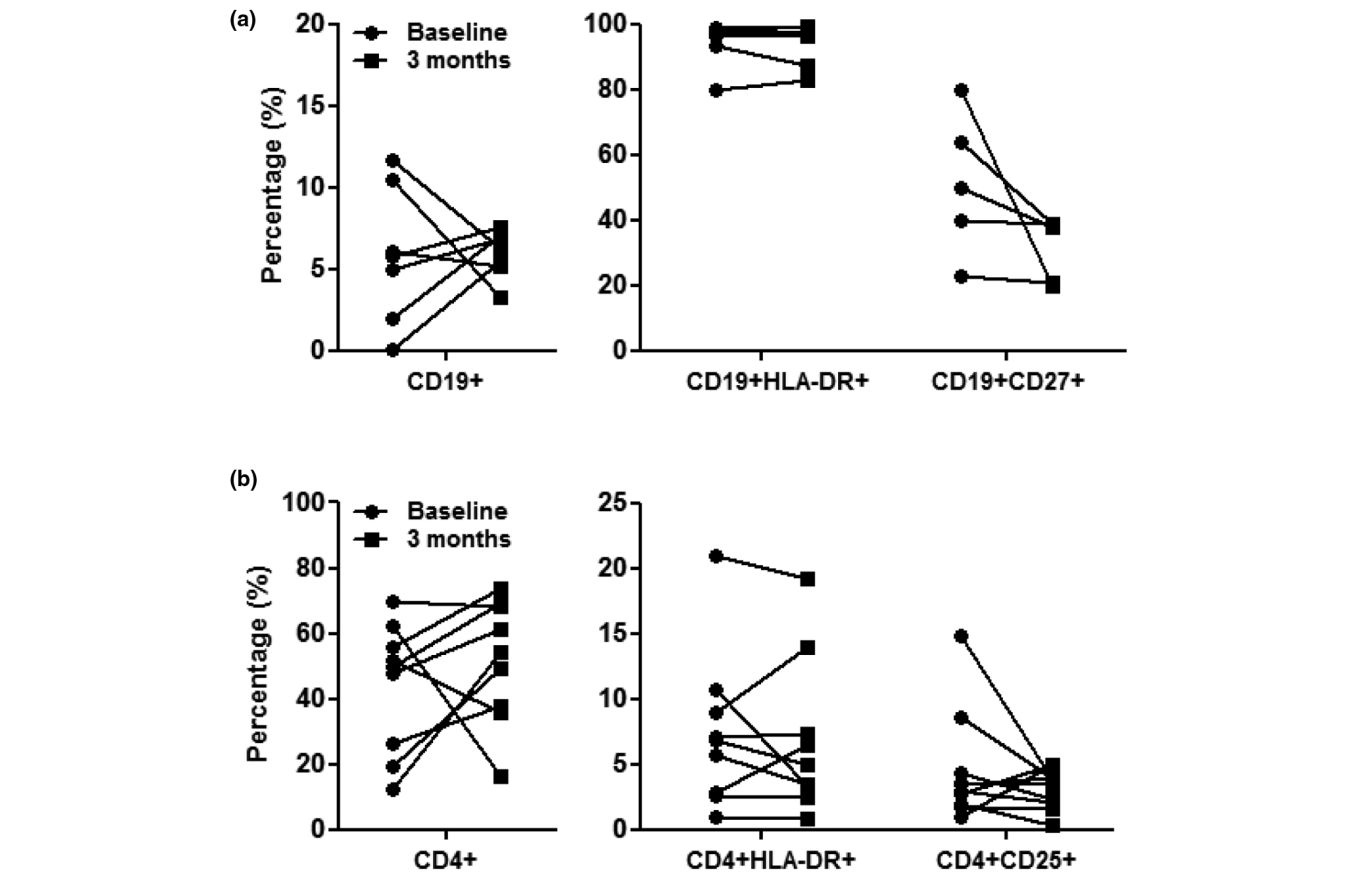
Effect of anti-TNF treatment on peripheral blood B cells in rheumatoid arthritis patients. **(a) **No effect of anti-TNF treatment on peripheral blood (PB) CD19^+ ^cells, CD19^+^HLA-DR^+ ^cells, and CD19^+^CD27^+ ^cells in rheumatoid arthritis (RA) patients (n = 7). B-cell depletion is specific to rituximab therapy. **(b) **Treatment with rituximab does not affect the proportion of total CD4^+ ^T cells, activated CD4^+^HLA-DR^+ ^T cells and CD4^+^CD25^+ ^T cells in the PB of RA patients.

To test whether B-cell depletion is specific to rituximab, we examined the effect of anti-TNF therapy on B cells in the PB of RA patients with active disease. After 12 weeks, anti-TNF-treated patients displayed only a marginal decrease in PB CD19^+ ^B cells from 7 ± 3% to 6 ± 1% (*P *= 0.670) (Figure 3a). Anti-TNF therapy had no effect on activated CD19^+^HLA-DR^+ ^cells (from 83 ± 14% to 94 ± 2%) and on memory CD19^+^CD27^+ ^B cells (from 44 ± 9% to 34 ± 4%, *P *= 0.171).

Finally, we examined the effect of rituximab on activated CD4^+ ^T cells by measuring the expression of the activation markers CD25 and HLA-DR by flow cytometry. We found no significant change in the percentage of CD4^+^CD25^+ ^T cells (from 11 ± 2% to 7 ± 1%, *P *= 0.563) and CD4^+^HLA-DR^+ ^T cells (from 7 ± 2% to 5 ± 1%, *P *= 0.667) 12 weeks after rituximab treatment (Figure 3b). Changes in CD4^+ ^T-cell markers did not correlate with the degree of B-cell depletion or response to therapy.

## Discussion

In the present study, we investigated the effects of rituximab on PB and BM lymphocyte subsets in patients with active RA. Rituximab effectively reduced CD19^+ ^B cells in the PB of patients; a less pronounced effect was observed in the BM. Importantly, rituximab therapy preferentially depleted activated CD19^+^HLA-DR^+ ^B cells in both compartments. The relative low clinical response rate in our population probably reflects the fact that these patients represent the most severe and refractory subset among our cohort, with several of them having failed multiple biologic therapies.

Expression and signaling through MHC class II molecules is important for effective antigen presentation and induction of co-stimulatory molecules on B cells [12]. Animal models of inflammatory arthritis support an important role of B cells in disease pathogenesis through a variety of mechanisms, including aberrant antigen presentation and activation of autoreactive T cells in the joint [13]. HLA-DR^+ ^B cells have been suggested to be important for B-cell-mediated T-cell activation; depletion of HLA-DR^+ ^cells could therefore represent an additional mechanism for the beneficial effects of rituximab in RA.

The BM is a primary lymphoid organ where B-cell differentiation and maturation occurs; BM stroma promote B-cell survival and protect B cells from depleting therapies [14,15]. In accordance with this observation, we found that rituximab therapy efficiently reduced PB CD19^+ ^B cells but had only a weak effect on BM CD19^+ ^B cells. The change in BM B cells did not correlate with clinical response to therapy or other disease parameters in the small patient number tested.

Previous studies have suggested the depleting effect of rituximab may be more pronounced in the PB than in inflamed tissues or lymphoid organs. Kavanaugh and colleagues found that - unlike PB B cells, which were profoundly (>95%) depleted after rituximab therapy - synovial B cells decreased but were not eliminated [16]. Similarly, in 24 RA patients who received rituximab, PB B cells were depleted at 4 weeks whereas synovial B cells were only moderately reduced and persisted in five patients [5]. Local tissue expression of B-cell survival factors (for example, B-cell activation factor belonging to the TNF family, stromal cell-derived factor-1, macrophage migration inhibitory factor) [17-19] might explain the relative resistance of B cells against the depleting effect of rituximab. Moreover, data both from animal models [20] and from humans [4,21] have shown that the effect of B-cell depleting therapies in lymphoid organs is variable. To this end, Teng and colleagues found that only 32% of RA patients had complete depletion of CD19^+ ^cells within the BM as compared with 100% in the PB [4]. Leandro and colleagues reported a variable degree of B-cell depletion within the BM [21].

In our cohort, four out of 11 patients had incomplete B-cell depletion in PB - a relatively high proportion compared with that observed by other studies. Nevertheless, resistance to rituximab has been described in autoimmune-prone animals. In MRL/*lpr *mice and in NOD mice it has therefore been shown that B cells are relatively refractory to depletion compared with nonautoimmune-prone strains [20,22]. In lupus-prone mice, high doses and extended duration of rituximab has been shown to overcome resistance to depletion; while in NOD mice, deficient FcγRI binding to IgG_2a _CD20 mAbs and reduced splenic monocyte numbers have been implicated in impaired B-cell depletion. The importance of FcγR in antibody-dependent cell-mediated cytotoxicity as a mechanism for B-cell depletion has been also shown in lupus patients treated with rituximab [23]. Whether these mechanisms are involved in reduced B-cell depletion in our patients needs to be addressed.

The significance of incomplete B-cell depletion at the sites of inflammation has not been fully determined, but investigators have suggested that disease progression - despite PB B-cell depletion - is probably due to survival of memory B cells, which expand in secondary lymphoid tissues and produce autoantibodies. In line with this hypothesis, Leandro and colleagues reported that patients who relapsed early after repopulation of PB B cells had a trend (*P *= 0.170) for a higher percentage of CD19^+^CD27^+ ^memory B cells compared with those patients that relapse later [24]. Roll and colleagues also performed immunophenotyping in the PB of RA patients who received rituximab, and reported a significantly higher proportion of IgD^+^CD27^+ ^memory B cells during B-cell recovery in nonresponders [25]. Moreover, Thurlings and colleagues found that the reduction of synovial tissue plasma cells, which are normally derived by memory B cells, between weeks 4 and 16 after treatment predicted clinical improvement at 24 weeks [5]. Accordingly, Möller and colleagues reported that low numbers of peripheral blood memory B cells (IgD^-^CD27^+^) correlated with good clinical responses in RA patients treated with rituximab [26].

Our data further support these findings since we observed a significant decrease in CD19^+^CD27^+ ^memory B cells not only in the PB but also in the BM of RA patients who subsequently responded to rituximab. In contrast, memory B cells in nonresponders remained stable or even increased in both compartments. Together these data suggest that analysis of memory B-cell subsets may provide important information on the efficacy and response to rituximab therapy, consistent with the notion that targeting cells with the memory phenotype is a key determinant for its efficacy. Whether persistence of PB and/or BM memory B cells is related to the lack of response after rituximab retreatment observed in RA patients who did not exhibit clinical improvement after the first treatment course [27] remains to be determined.

Previous studies have indicated that rituximab may affect T-cell responses in systemic lupus erythematosus patients through downregulation of activation and co-stimulatory molecules (for example, CD69, CD154). This effect was attributed to decreased antigen-presenting function of B cells after rituximab treatment. We assessed the population of activated CD4^+^CD25^+ ^and CD4^+^HLA-DR^+ ^T cells, but we found no consistent changes according to response to therapy. Similarly, Thurlings and colleagues found no effect of rituximab therapy on PB CD4^+ ^or CD8^+ ^T cells of RA patients [5], whereas Leandro and colleagues reported a decrease only in a small subpopulation of CD3^+^CD20^+ ^cells while the total CD4^+ ^subset and the activated CD4^+ ^subset remained unaffected [24]. Nevertheless, an effect on T-cell activation/function cannot be excluded. Therefore, although B-cell depletion in an animal study had no effect on T-cell subsets and activation markers, it impaired adaptive and autoreactive CD4^+ ^T-cell activation [28].

Of note in small uncontrolled trials of systemic lupus erythematosus patients, it has been reported that rituximab reduces activated CD4^+ ^cells in parallel with B cells. To this end, Sfikakis and colleagues reported that lupus patients responding to rituximab downregulated CD40 ligand, CD69 and HLA-DR expression on CD4^+ ^cells [29], while Tokunaga and colleagues reported downregulation of CD40 ligand, inducible costimulatory molecule (ICOS) and CD69 on CD4^+^-positive cells in patients (n = 3) with active systemic lupus erythematosus [30,31]. Whether the effect of rituximab on activated T cells is disease specific remains to be seen.

Response rates according the European League Against Rheumatism criteria (45% good and moderate responders) were moderate in our cohort. Nevertheless, this was a group of RA patients who were highly active refractory to previous treatment. All but one patient had failed in treatment with anti-TNFα agents (up to three agents). Although we found no correlation between clinical response and the degree of peripheral B-cell reduction, incomplete B-cell depletion (four out of 11 patients) could potentially be one of the reasons for lower clinical responses.

## Conclusions

Rituximab effectively reduced PB CD19^+ ^B cells in RA patients whereas its effect was less pronounced in the BM. Activated CD19^+^HLA-DR^+ ^cells were significantly reduced in both compartments. Importantly, clinical response was associated with the persistence of memory B cells in both compartments.

## Abbreviations

BM: bone marrow; DAS28: disease activity score of 28 joint counts; IL: interleukin; mAb: monoclonal antibody; PB: peripheral blood; RA: rheumatoid arthritis; TNF: tumor necrosis factor.

## Competing interests

The authors declare that they have no competing interests.

## Authors' contributions

MN carried out the immunological studies. GK participated in the design and conduction of the study and drafted the manuscript. PS participated in the design of the study, performed the statistical analysis of the data and drafted the manuscript. GB performed the statistical analysis of the data and drafted the manuscript. EP carried out the immunological studies. AR carried out the immunological studies and was involved in patient care. HK was responsible for analysis of flow cytometry experiments. HAP participated in the design of the study and drafted the manuscript. HK participated in the design and conduction of the study, was involved in patient care and reviewed the manuscript. DTB conceived of the study and participated in its design and coordination.
